# Long-term follow-up after anterior cruciate ligament reconstruction using a press-fit quadriceps tendon-patellar bone autograft

**DOI:** 10.1186/s12891-018-2271-8

**Published:** 2018-10-12

**Authors:** Alexander Barié, Michael Köpf, Ayham Jaber, Babak Moradi, Holger Schmitt, Jürgen Huber, Nikolaus Alexander Streich

**Affiliations:** 1 Clinic for Orthopedics and Trauma Surgery, Center for Orthopedics, Trauma Surgery and Spinal Cord Injury, Heidelberg University Hospital, Schlierbacher Landstrasse 200a, 69118 Heidelberg, Germany; 2 Center for Joint Surgery and Sport Injuries, Sportopaedie Heidelberg, Clinic St. Elisabeth Heidelberg, Max Reger Strasse 5-7, 69121 Heidelberg, Germany; 3 Center for Orthopedics and Sports Trauma Surgery, Atos Clinic Heidelberg, Bismarckstraße 9-15, 69115 Heidelberg, Germany

**Keywords:** Knee, Anterior cruciate ligament, Reconstruction, Quadriceps tendon, Press-fit technique

## Abstract

**Background:**

The use of a quadriceps tendon-patellar bone (QTPB) autograft provides an alternative procedure in primary reconstruction of the anterior cruciate ligament (ACL). Using the press-fit technique for femoral fixation and knotting over a bone bridge as well as additional spongiosa filling for tibial fixation can completely eliminate the need for fixation implants. The objective of this study was to evaluate the long-term clinical, functional and radiological results of this operating method.

**Methods:**

Sixty-nine patients (27 female-42 male) were included in this study. Fifty-seven patients (83%) received a comprehensive follow-up review after an average period of 7.5 years (range: 7–8.7). All other patients were surveyed by telephone. Six patients (9%) suffered a re-rupture of the ACL graft caused by a new related trauma and were therefore excluded from the statistical analysis.

**Results:**

Of all patients, 98% were satisfied with the operation. Normal or almost normal results were recorded in the subjective IKDC scores form by 88% of the patients. The Lysholm score demonstrated very good and good results in 83% of the patients. Only 1 patient reported minor complaints in the donor area. Seven (12%) patients developed Cyclops syndrome with limited knee extension. This complication was treated arthroscopically within the first year postoperatively. Their results on follow-up were not worse than the results of the patients without Cyclops syndrome.

Regarding the 57 patients who received a comprehensive evaluation, the stability test with the KT-1000 Arthrometer yielded a difference of less than 3 mm in the contralateral comparison for 89% of the operated knees. The pivot-shift test was normal in 79% and almost normal in 21%. In the Single-leg Triple Hop Test, patients achieved an average of 98% of the hopping distance attained with the contralateral leg. The radiological examination revealed a slight deterioration in the Kellgren-Lawrence Score in 2 patients.

**Conclusion:**

The ACL reconstruction using the QTPB autograft performed with the press-fit technique leads to good results in comparison with published results of established procedures for primary ACL surgery using other autografts. Further investigations should involve comparative studies with the objective of providing evidence-based, individually adapted therapy for ACL rupture.

**Electronic supplementary material:**

The online version of this article (10.1186/s12891-018-2271-8) contains supplementary material, which is available to authorized users.

## Background

Anterior cruciate ligament (ACL) reconstruction is considered to be the gold standard in ACL insufficient knees. The aim of the surgery is to reduce the onset of secondary degenerative changes as well as to restore the ability to perform sport activities on a level comparable to that prior to incurring the injury. Due to the high incidence of ACL ruptures in the young and active patient population [1], there exists a growing interest in the advancement of the surgical technique. The goals of improvement include reducing donor site morbidity, providing an adequate knee function, as well as maintaining knee stability with a low complication rate on long-term follow-up.

The femoral press-fit fixation in ACL reconstruction was originally developed and described by Hertel in 1987. A following tibial press-fit fixation technique was then presented in 1989 [2]. Boszotta and colleagues described the procedure using an arthroscopic approach in 1997 [3]. Afterwards, several authors reported their experience with this surgical technique [4–8]. Advantages of the press-fit fixation include direct bone-to-bone healing, accelerated rehabilitation, absence of hardware related complications or allergic reactions, less costs and the ease of revision procedures since there is no metal removal or tunnel enlargement [9, 10].

The use of a quadriceps tendon-patellar bone (QTPB) autograft for anterior cruciate ligament (ACL) reconstruction was first described by Blauth in 1984 [11]. As a result, the suitability of the quadriceps tendon graft for ACL reconstruction was compared with the patellar tendon graft. Anatomical studies showed that a 10 mm wide tendon strip from the middle third of the quadriceps tendon has a significantly higher cross-sectional area than a 10 mm wide patellar tendon strip and that the attachment surface at the patella was significantly bigger in the quadriceps tendon graft with bone block [12]. Experimental attempts showed an equivalent or higher tear resistance for the quadriceps tendon graft [13]. In spite of these good experimental results, either hamstring tendon autografts or patellar tendon autografts remained to be the gold standard for ACL reconstruction [14]. In two recent reviews, a total of 24 clinical studies with a mid-to long-term follow-up using the QTPB autograft were identified and evaluated, leading to the conclusion that this graft is a safe alternative with good results and low donor site morbidity [15, 16]. The published results of ACL reconstruction using QTPB anchored using a press-fit technique with 1-year follow-up show promising results [17, 18]. The long-term outcome of this technique needs further evaluation. The objective of this study was therefore to examine the clinical results with a long term follow-up after ACL reconstruction with the QTPB autograft.

## Methods

### Study population and follow-up

The survey retrospectively included all patients operated on with a minimum of 7 years and a maximum of 9 years before the start of the study. Sixty-nine patients had received an arthroscopic reconstruction of the ACL using a QTPB autograft with hardware-free fixation and met the strict inclusion and exclusion criteria (Table 1). All patients were operated on by one senior surgeon (JH). Twenty-seven (39%) of the patients were female and 42 (61%) were male. The average age on operation day was 27 years (range: 14 to 43 years).

**Table 1:** Criteria of patient selection for this study

Inclusion criteria	Exclusion criteria
Primary ACL rupture	Previous operations on both knee joints
Maximum 18 months between injury and operation	Injuries of the lateral collateral ligament and the posterior cruciate ligament
Maximum age at operation 45 years	Chondromalacia greater that grade II according to Outerbridge
Age of at least 18 years on investigation	Meniscal repair
Activity level before the injury ≥4 in the Tegner Score	Instability of the contralateral knee joint

Six patients (9%) suffered a re-rupture of the ACL graft during the follow-up period as a result of a new trauma; these patients received a revision ACL reconstruction using a different surgical technique and were therefore excluded from the statistical analysis.

After the retrospective inclusion of the patients, we prospectively performed the collection of the data. A total of 6 patients (9%) did not attend for physical examination but all of them were reached and have completed the subjective surveys on the telephone.

The period between injury and operation was on average 4.3 months (range: 8 days to 15 months). The investigation was carried out after a mean follow-up interval of 7.5 years (range: 7.0 to 8.7) by two experienced orthopedic specialists (AB, NAS) who were not involved in the primary operation.

### Mechanism and level of injury

Sixty-four (93%) patients incurred ACL injuries during sports: football (*n* = 23, 36%), skiing (*n* = 15, 24%), handball (*n* = 12, 18%), basketball (*n* = 5, 8%), tennis (*n* = 3, 5%), volleyball (n = 2, 3%), rugby (n = 2, 3%), fistball (n = 1, 1.5%), gymnastics (n = 1, 1.5%). Five patients (7%) were injured during the course of everyday physical activities. Additional lesions were identified with meniscus damage in 24 (35%) patients, cartilage damage in 8 (12%) patients and meniscus damage and cartilage damage in 16 (23%) patients. Two patients (3%) had a combination of ACL rupture, rupture of the medial collateral ligament, tear of the outer meniscus and cartilage damage. Only 19 (27%) patients had an isolated ACL rupture. Since meniscus repair was an exclusion criterion, only patients with partial meniscus resection (*n* = 34, 50%) or stable meniscus lesions which were not addressed (*n* = 8, 12%), were included. Interventions on the cartilage were not carried out in the case of articular cartilage damage °I (n = 8, 12%) and °II (*n* = 15, 22%). Higher grade cartilage damage led to exclusion from the study [19].

### Surgical procedure

**Fig. 1:**
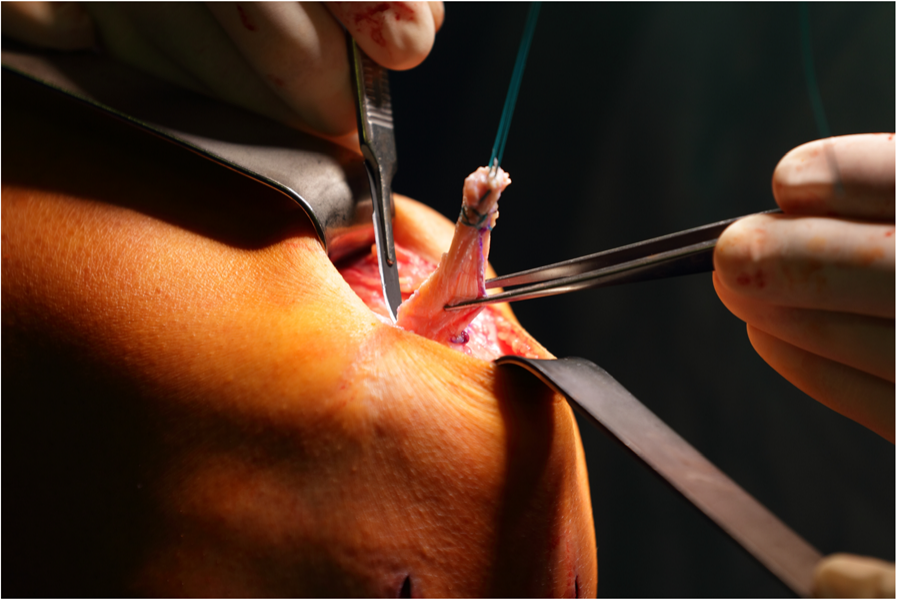
A 5 cm long, 1 cm wide and 6 to 8 mm-thick strip of the quadriceps tendon is dissected out and reinforced with Mersilene threads

**Fig. 2:**
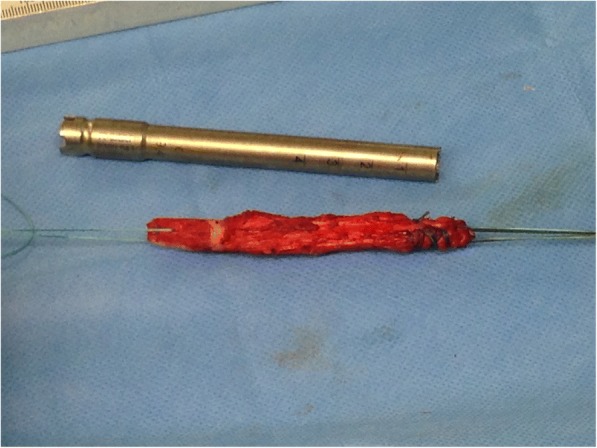
The armed transplant before implantation

**Fig. 3:**
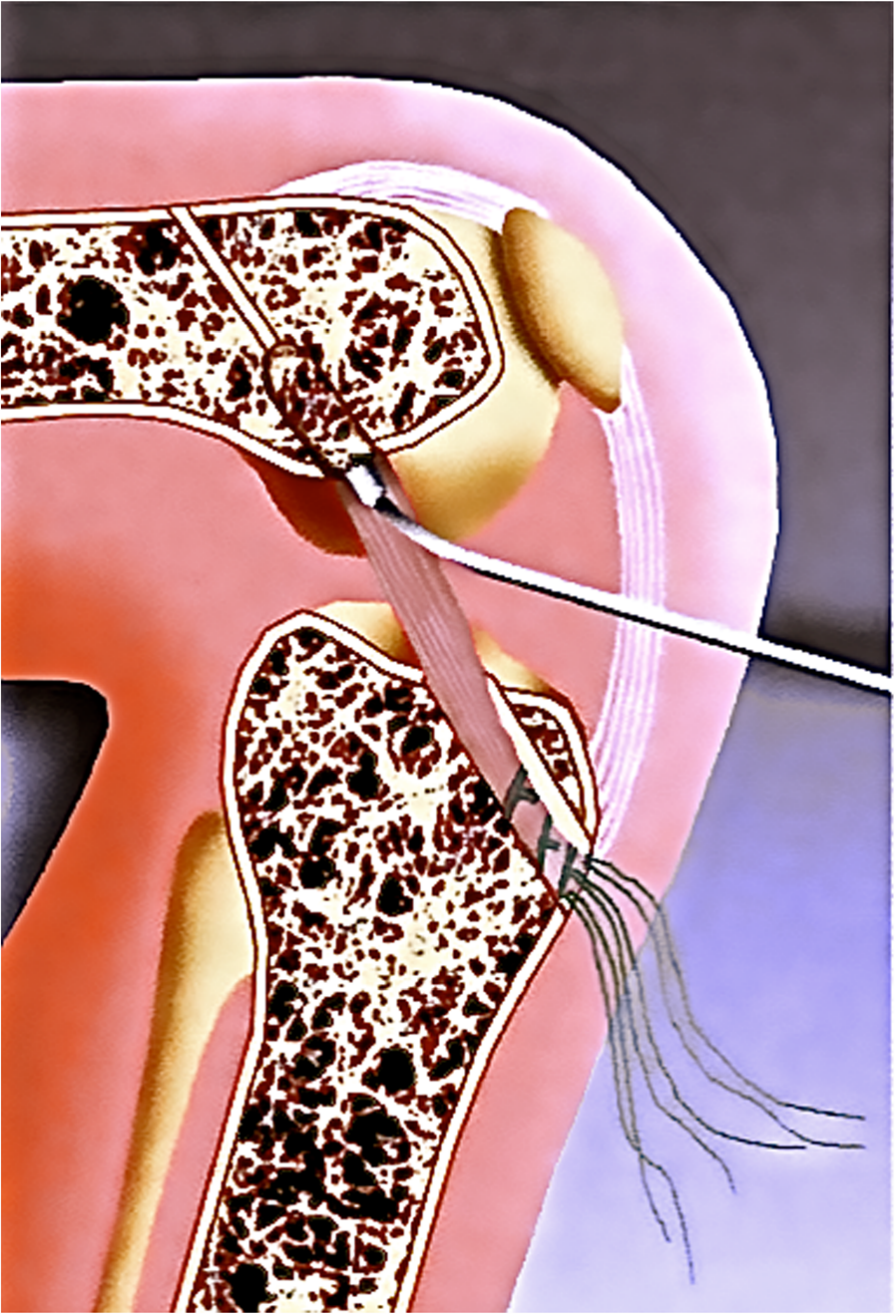
Diagram showing the press-fit fixation of the patellar bone cylinder in the femoral tunnel

**Fig. 4:**
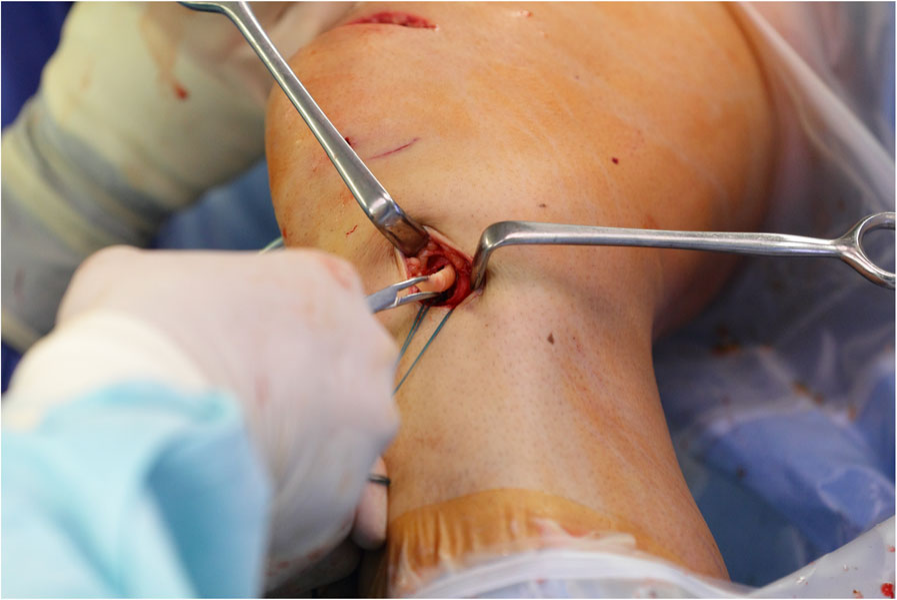
Spongiosa transplantation filling up the tibial tunnel

ACL reconstruction was performed using a technique in which fixation of the graft and the bony filling for the tunnels can be done without the use of hardware. This is made possible by using oscillating hollow burrs (Additional file 1: Figure SA) and a special asymmetrically shaped spongiosa compactor. When harvesting the graft (Fig. 1 / Additional file 2: Figure SB), the diameter of the cylindrical patellar bone block can thereby be precisely defined to 9.4 mm (Fig. 2). The femoral press-fit anchoring is then done by using the bone block to plug the 8 mm femoral tunnel that has been drilled and slightly dilated (Fig. 3). A hollow burr is also used to create the tibial tunnel so that a spongiosa cylinder can be harvested. The tibial fixation is carried out as a hybrid technique. Blocking the tendon in the tibial tunnel with the harvested bone cylinder (Fig. 4) leads to rapid healing of the bone and the knotting of the threads reinforces the quadriceps tendon over a bone bridge (Additional file 3: Figure SC), ensuring primary stability. A detailed description of the individual steps to the surgical procedure and the standardized rehabilitation were described in 2013 [20].

### Questionnaires / subjective activity level

A general questionnaire surveyed the patients’ data for details of the trauma mechanism and the subjective satisfaction of the patient. Patients specified their satisfaction with the result of the operation on a Visual Analog Scale (VAS) of 0 to 10 [21].

The sporting activity level of the patients was surveyed before the injury and at the time of the follow-up investigation using the Tegner Score of 0 to 10. Level 0 corresponds to an inability to work, level 5 corresponds to the ability to do more intensive physical activities such as cross-country skiing, cycling and jogging two times a week, whereas level 10 corresponds to the ability to play football on a national or international level [22]. The Lysholm Score served to evaluate the subjectively perceived impairment of knee-joint function. It is scored using a point system that evaluates the knee joint in eight everyday stress situations. By adding the points a maximum of 100 points can be achieved. A score of 91–100 points refers to a very good knee function, 84–90 points is good, 65–83 points is moderate and 0–64 points refers to a bad knee function [23]. The Form of the IKDC Score 2000 (International Knee Documentation Committee) for subjective assessment of the knee in everyday and sporting activity was also assessed. It includes seven questions about the symptoms and two questions on activities and function of the knee joint. The result is a point value in which category A (90–100 points) represents a normal knee function, category B (80–89 points) is nearly normal, category C (70–79 points) is abnormal and category D (0–69 points) represents a strongly abnormal knee function [24].

### Clinical investigation

The clinical investigation was carried out using the Form for investigation of the knee for the IKDC Score 2000 [24]. The instrumental measurement of the anterior-posterior stability was carried out using the KT-1000™ Knee Ligament Arthrometer® manufactured by MEDmetric® Corporation (http://www.medicalproductguide.com/companies/1364/medmetric_corp, MEDmetric® Corporation, 7542 Trade Street, San Diego, CA 92121; Patent no. 4,583,555) and a tension force of 134 N. The clinical investigation was supplemented by thigh circumference measurements and the Single-Leg Triple-Hop Test [25]. Two measurements of the thigh circumference were done, 10 cm and 20 cm proximal to the joint line. Järvelä and colleagues showed a highly significant correlation (*p* < 0.001) between thigh measurement and the isokinetic strength test for knee extension [26].

### Radiological investigation

Osteoarthritis stage was evaluated according to Kellgren-Lawrence classification [27]. Assessment of the tunnels involved X-rays of the knee taken immediately postoperatively in three planes (exercise X-ray according to Rosenberg in the posterior-anterior beam path at 25° knee bend, lateral view at 30° flexion standing and 1 patella-tangential view). The same views of X-rays were taken on follow-up and were compared with the postoperative X-rays.

### Subgroup analysis

We further performed a subgroup analysis in order to investigate whether different clinical variables had an impact on the postoperative results. For this purpose, the patient collective was divided into two groups and analyzed statistically. The divisions were made according to sex (Male / Female), by age (< 30 years old on operation day / ≥ 30 years old on operation day), by Body Mass Index (< 25 / ≥ 25), period between injury and surgery (< 3 months ≥3 months), Tegner score before injury (< Level 8 / ≥ Level 8), accompanying injuries in the form of meniscal injuries (presence of meniscal injury / no meniscal injury), cartilage damage (presence of cartilage damage / no cartilage damage), difference in KT-1000 measurement (≤ 2 / > 2), result of the pivot shift test (no pivoting / pivoting), measurement of the thigh circumference (thigh circumference of operated leg ≥ thigh circumference of contralateral side) / thigh circumference of operated side < thigh circumference of contralateral side), and postoperative complications (patients without cyclops or other complications / patients with cyclops or other complications).

### Statistical analysis

The entire data input and evaluation was carried out using the program IBM® SPSS® version 24. The normal distribution test was performed by the Kolmogorov-smirnoff, respectively by the Shapiro-Wilk test and the variance homogeneity by the Levine test. For the comparative statistics of the subgroups, statistical significance was calculated for the metric scaled variables according to distribution and variance homogeneity by the t-test or the Mann-Whitney U-test. For the ordinal scaled variables, the evaluation was done using the Mann-Whitney U-test or by correlation with the spearman rank coefficient. Nominal scaled variables were also evaluated by the Mann-Whitney U-test or by cross-tabulation using the Chi-square test or fisher’s exact test. The comparison of two measurement points recorded at two different times was done by the Wilcoxon test. For all the tests used, a *p*-value of less than 0.05 was considered as significant.

## Results

### Complications and revision interventions

There were no intraoperative complications. One patient (1.5%) had a wound infection postoperatively which was treated conservatively with antibiotics. One patient (1.5%) had a deep vein thrombosis which was treated with anticoagulant therapy. Seven patients (12%) experienced limited knee extension postoperatively. A consequent magnetic resonance imaging (MRI) scan identified the development of a Cyclops in all 7 cases. An arthroscopic operation with resection of the Cyclops and a notchplasty was carried out in all cases after approximately 6 months. Thereafter all patients achieved full extension of the knee joint. The 7-year results of the patients with complications differed neither in the subjective nor the objective parameters from the patients without complications.

### Subjective questionnaires

On Follow up, 42 patients (74%) were very satisfied (VAS 9–10), 11 patients (19%) were satisfied (VAS 6–8), 3 patients (5%) were moderately satisfied (VAS 3–5) and 1 patient (2%) was dissatisfied (VAS 0–2).

The median level of physical activity measured by using the 11-stage Tegner Score (0–10) fell significantly from 7 (range: 4–9) before the injury to 6 (range: 1–9) at the time of the follow-up investigation (*p* = 0.001). On follow-up, 36 (63%) patients achieved at least the same activity level as before the injury and 21(37%) patients remained one to five stages below that.

The average value for the Lysholm Score at the follow-up examination was 92 ± 10.6 points (range: 60–100). Forty-two patients (74%) rated their knee function as very good (91–100 points), 5 (9%) had good knee function (84–90 points), 7 (12%) had moderate knee function (65–83 points) and 3 (5%) had poor knee function (≤ 64 points).

The average value of the scores for subjective assessment of knee-joint function for everyday activities and in sport (IKDC 2000 Form) was 90 ± 9.9 points (range: 47–100). Thirty-seven patients (65%) of the patients had normal knee function (90–100 points). Thirteen (23%) patients had almost normal knee function (80–89 points), 5 (9%) patients had abnormal knee function (70–79 points) and 2 (3%) patients had significantly abnormal knee function (≤69 points). The evaluation of question 9 of the subjective IKDC 2000 Form revealed no difficulties in kneeling or squatting in any of the patients.

### Clinical investigation

Table 2 shows the results of clinical investigations using palpation and instruments based on the IKDC 2000 Form for clinical investigation of the knee. The posterior-anterior translation was measured using the KT-1000 Arthrometer and yielded an average value of 6.7 ± 1.7 mm (range: 2 to 11 mm) for the unoperated knee and an average value for the operated knee of 7.5 ± 1.7 mm (range: 3 to 12 mm). This increased sagittal translation of the operated knee from an average of 0.9 ± 1.3 mm (range: -2 mm to 4 mm) was statistically significant (*p* = 0.004). The circumference measured for the thigh 10 cm proximal to the base of the patella gave an average value for the operated knee of 46 ± 4.2 cm (range: 39-58 cm) and 47 ± 4.7 cm (range: 39-65 cm) for the unoperated knee. Twenty cm proximal to the patella yielded an average of 55 ± 4.7 cm (range: 45-69 cm) for measurement of the operated knee and 56 ± 4.7 cm (range: 47-75 cm) for the unoperated knee. However, the lower circumference for the operated thigh was not significant for both values (*p* = 0.408). In the Single-leg Triple-Hop Test, the hopping distance achieved in three consecutive single-leg hops was measured and the side-to-side difference was determined. Two patients did not want to take part in this test. The average hop distance for the operated leg was 416 ± 91 cm (range: 225-635 cm) and for the unoperated leg was 427 ± 93 cm (range: 200-635 cm). The difference was not statistically significant (*p* = 0.570). Furthermore, only one patient (1%) reported hypaesthesia (sensitivity to touch) at the donor site, none reported anterior knee pain. Meniscus tests were abnormal in 1 patient. This patient also had the worst functional results.

**Table 2:** Results of the clinical, instrumental and radiological investigations based on the IKDC 2000 Form for knee evaluation

*N* = 57	Number of patients (percent of patients)			
Investigation	Normal	Almost Normal	Abnormal	Significantly abnormal
1. Effusion	56 (98%)	1 (2%)	–	–
2. Movement deficit				
Extension deficit	57 (100%)	–	–	–
Flexion deficit	55 (96%)	2 (4%)	–	–
3. Ligament examination				
KT 1000 Arthrometer	51 (89%)	6 (11%)	–	–
Lachmann Test (manual)	50 (88%)	7 (12%)	–	–
Posterior Drawer Test	57 (100%)	–	–	–
Valgus stress	57 (100%)	–	–	–
Varus stress	57 (100%)	–	–	–
Pivot-shift Test	45 (79%)	12 (21%)	–	–
4. Crepitation	44 (77%)	8 (14%)	5 (9%)	–
5. Donor site morbidity	56 (98%)	1 (2%)	–	–
6. Joint space narrowing on X-ray				
Directly postoperative (*n* = 54)	54 (100%)	–	–	–
On follow-up (*n* = 54)	52 (96%)	2 (4%)	–	–
7. Single-leg Triple-Hop Test (*n* = 55)	49 (89%)	6 (11%)	–	–

### Radiological investigation

On follow-up, three patients were pregnant. As a result, the radiological examination could only be carried out in 54 (78%) patients. The X-rays were taken directly postoperatively and on follow-up for these patients. Assessment of osteoarthritis according to Kellgren-Lawrence showed deterioration by one stage in two patients (Table 2). The assessment of the drill tunnel diameter provided radiological evidence in all patients for complete bony filling of the femoral tunnel and almost complete bony filling of the tibial tunnel. No tunnel-widening occurred in any patients. None of the patients experienced a dislocation of the femoral bone block or dislocation of the tibial spongiosa cylinder.

### Subgroup analysis

The following differences were revealed in the subgroup analysis:

Older patients had a greater loss of activity level in the Tegner Score than younger people (*p* = 0.013). Patients with a longer waiting time between injury and surgery achieved a poorer score in the Pivot-Shift Test than patients with a shorter waiting time (*p* = 0.017). Patients who had meniscus injuries achieved a poorer score in the KT-1000 stabilization measurement than patients without a meniscus injury (*p* = 0.033) and their difference in the thigh circumference was greater (*p* = 0.028). Patients who had cartilage damage had a knee flexion deficit more frequently than patients without cartilage damage (*p* = 0.009). No significant differences in outcome could be identified in regards to the following parameters: sex (*p* = 0.74), Body mass index (*p* = 0.073), Tegner level of sporting activity before the injury (*p* = 0.334), and complications (*p* = 0.366).

## Discussion

The objective of this study was to evaluate the long-term outcome of the surgical technique for ACL reconstruction with a QTPB autograft fixated without the use of hardware. To date, only short-term results with a follow-up after 12 months are available with good clinical outcomes [17, 18]. Clinical studies with a long-term follow-up after ACL reconstruction using bone-patellar tendon-bone autograft using a press-fit fixation reported good results. As far as the authors are aware, only results with a maximum average follow-up of 5.6 years [28] are available for other techniques using hardware for fixation of the QTPB autograft.

Critics have expressed doubts about the stability of the fixation technique without the use of hardware fixation. In the biomechanical studies, the femoral press-fit fixation used here possessed adequate primary stability with ultimate load to failure at least equal to results for interference screws [29, 30]. The tibial hybrid fixation with bone bridge and spongiosa filling also demonstrated the same tear strengths under experimental conditions as interference-screw fixation [31]. This clinical study identified no complication in any patient which could be assessed as a consequence of the fixation technique. No tunnel expansion occurred comparable to that reported with other fixation materials [32]. This is comparable to other studies that report a decreased rate of tunnel expansion on follow-up after ACL-Reconstruction using this procedure [10]. Also in the 6 patients with re-rupture, the drill tunnels were filled with bony material so that a revision operation on one side without the need for spongioplasty was possible.

The clinical stability measurements yielded normal values for 88% of the patients in the Lachmann Test, for 89% in the KT-1000 Arthrometer measurement and for 79% in the Pivot-Shift Test. These values were within the range of values obtained for other techniques. It is described that normal values were obtained for the patellar-tendon graft with the Lachmann Test in 76–100% and with the Pivot-Shift Test in 81–100% of the patients. For the hamstring tendon graft, 64–100% of the patients obtained normal values with the Lachmann Test and 72–100% with the Pivot-Shift Test [33].

The average value of 92 points in the Lysholm Score was also within the range of the specified values for the patellar tendon graft (91–93) and for the hamstring tendon graft (80–94). Eighty-eight percent of the patients achieved a normal or almost normal value in the subjective IKDC 2000 Form. Comparable studies published 48–97% for the patellar tendon graft and 50–97% for the hamstring tendon graft [33].

All the parameters for knee-joint laxity and function that have been assessed were within the range of values similar to that achieved in the patellar tendon and hamstring tendon grafts that are most frequently used and are often designated as the Gold Standard [34, 35]. The primary targets of the ACL operation could therefore be achieved using this surgical procedure with good dependability even after 7.5 years.

Fractures of the patella and tears of the quadriceps tendon are specific complications giving rise to concerns when using the QTPB autograft [36]. However, these specific complications did not occur in the previous reports with short-term follow-up [17, 18] and also not in this study. It is therefore questionable as to whether these potential complications are of clinical relevance.

A complication brought to light in this study is the development of Cyclops syndrome in 12% of the patients. High rates of Cyclops syndrome were also specified for the patellar tendon graft at 11% [37] and 7% [3]. The rates for the hamstring tendon graft are generally lower at up to 3% [38]. Ultimately, the reason for this development is not clear. One possible and reasonable explanation is that the quadriceps tendon graft and the patellar tendon graft have a rougher texture than the hamstring tendon and fibers sloughing off during the process of ligamentization may lead to growths in the area of the tibial ACL insertion. MRI diagnostics is important in patients with persistent knee extension deficit followed by surgical revision using arthroscopic debridement of the hypertrophied tissue and if necessary supplementary notch graft. Full knee extension was achieved for all the patients participating in this study and there were no negative effects on the knee function at final follow-up. This confirms the previous clinical experience that there is generally no recurrence after the mechanical obstruction has been removed [39].

Most grafts do not seem to be significantly different on long-term follow-up. This is presumably a result of the intraarticular ligamentization process that all grafts undergo after reconstruction. This remodeling eventually leads to a ligamentous “ACL-like” structure which histologically resembles a normal ACL. Only ultrastructural differences regarding collagen fibril distribution persist [40]. The ligamentization process has been mainly described in the patellar tendon graft and the hamstring tendon graft. Studies that examine the ligamentization process of the QTPB autograft are lacking.

Measurements of thigh circumference were carried out to investigate muscular weakness. These revealed no significant differences between the operated and unoperated leg. The Single-Leg Triple Hop Test also demonstrated no significant statistical differences. Eighty-nine percent of the patients achieved at least 90% of the hop distance achieved with the unoperated leg. Essentially similar results of 80–93% were published in the literature for hamstring tendon graft and patellar tendon graft [38].

The donor site morbidity of the QTBP autograft is frequently the focus of discussion. The donor site morbidity in this study was very low (2%). This rate is significantly lower compared to the bone-patellar tendon-bone autograft using the same fixation technique [7, 8, 41]. The previous results for the quadriceps tendon graft using hardware fixation also revealed a low rate of anterior knee pain [15, 16].

The subgroup analysis confirmed the known negative influencing factors of the associated meniscus tear and the associated cartilage injury. An interesting finding was the fact that patients with a short interval between injury and operation performed significantly better in tests for rotational stability than patients with an interval of more than 2 months. One possible explanation for this could be that the anatomical footprints of the ACL may be easier to identify when the operation is done at an early stage. This makes it easier to carry out the anatomical reconstruction.

A limitation of this study is the lack of a control group. It is important to note for purposes of radiological evaluation that the X-ray images were not available in digital form and the assessment of the width of the joint space and tunnels therefore represent subjective assessments by the two follow-up investigators. However, the assessments by the two follow-up investigators were separate and blinded, and they were identical. The methods used to assess muscular weakness are hardly valid. An attempt to establish isokinetic power measurement in this study failed. A second investigation date would have been required at a different site and most patients rejected this.

## Conclusions

The results of our evaluation suggest that this surgical method using a QTPB autograft with hardware-free graft fixation provides a reliable alternative for reconstruction of the anterior cruciate ligament. Advantages of press-fit fixation include avoidance of complications related to hardware fixation, direct bone-to-bone healing, cost effectiveness compared to other fixation techniques, and ease of revision surgery if needed. Furthermore, the use of the quadriceps tendon autograft showed lower donor site morbidity compared to other grafts. However, a complication in the form of Cyclops syndrome must always be considered.

Further investigation should involve short-term studies that describe the influence of QTPB autograft on short-term disability, second injury rates and quadriceps strength deficits. Comparative studies with different surgical techniques could focus on assessing muscular regeneration in the context of rehabilitation.

## Additional files

Additional file 1: **Figure SA.** Two oscillating hollow burrs are used to harvest the patellar bone block and to create the tibial tunnel. A curved plunger is used to press the patellar bone block in the femoral tunnel. (JPG 2510 kb)

Additional file 2: **Figure SB.** The oscillating hollow burr is pushed over the free tendon to saw out a cylinder, 22 mm in length, of the ventral upper pole of the patella. (JPG 3338 kb)

Additional file 3: **Figure SC.** Knotting the Mersilene threads over the tibial bone bridge. (JPG 4293 kb)
