# Supplementary figures and images for: Long-term follow-up after anterior cruciate ligament reconstruction using a press-fit quadriceps tendon-patellar bone autograft
Source: BMC Musculoskelet Disord. 2018 Oct 12;19:368. doi: 10.1186/s12891-018-2271-8 (PMC6186094; doi:10.1186/s12891-018-2271-8)

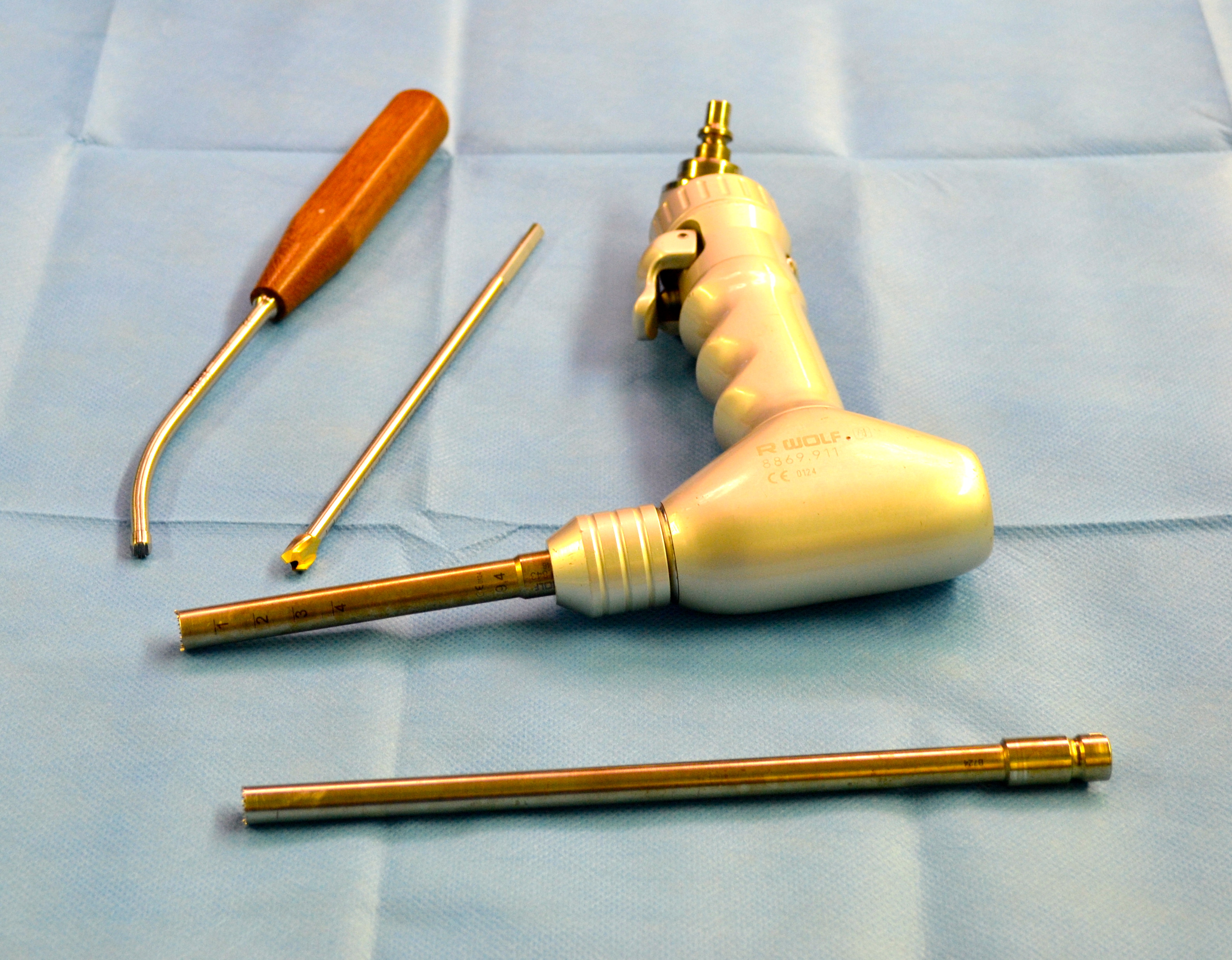

Supplement: Supplementary file 1 — Figure SA. Two oscillating hollow burrs are used to harvest the patellar bone block and to create the tibial tunnel. A curved plunger is used to press the patellar bone block in the femoral tunnel. (JPG 2510 kb) [file 12891_2018_2271_MOESM1_ESM.jpg]

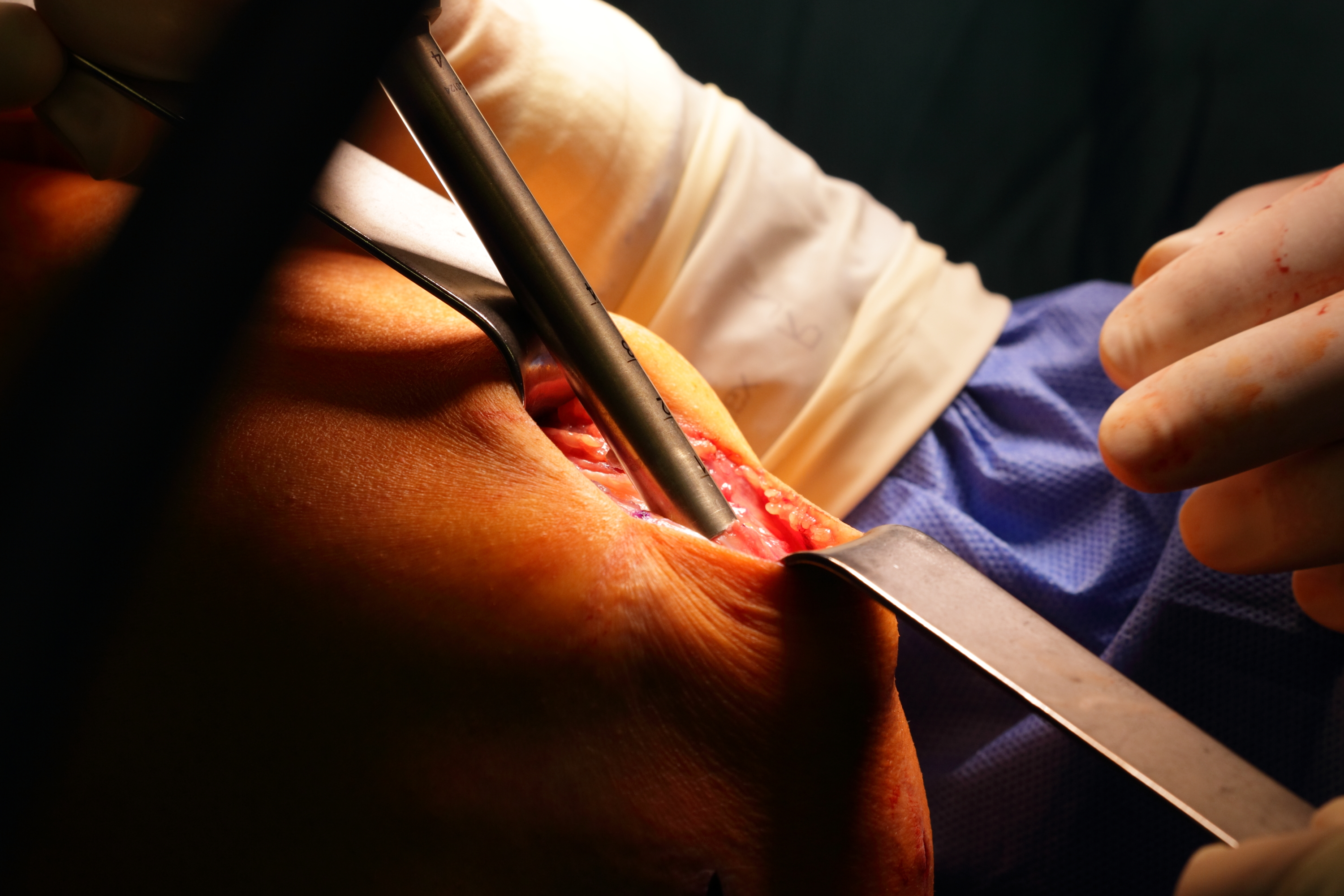

Supplement: Supplementary file 2 — Figure SB. The oscillating hollow burr is pushed over the free tendon to saw out a cylinder, 22 mm in length, of the ventral upper pole of the patella. (JPG 3338 kb) [file 12891_2018_2271_MOESM2_ESM.jpg]

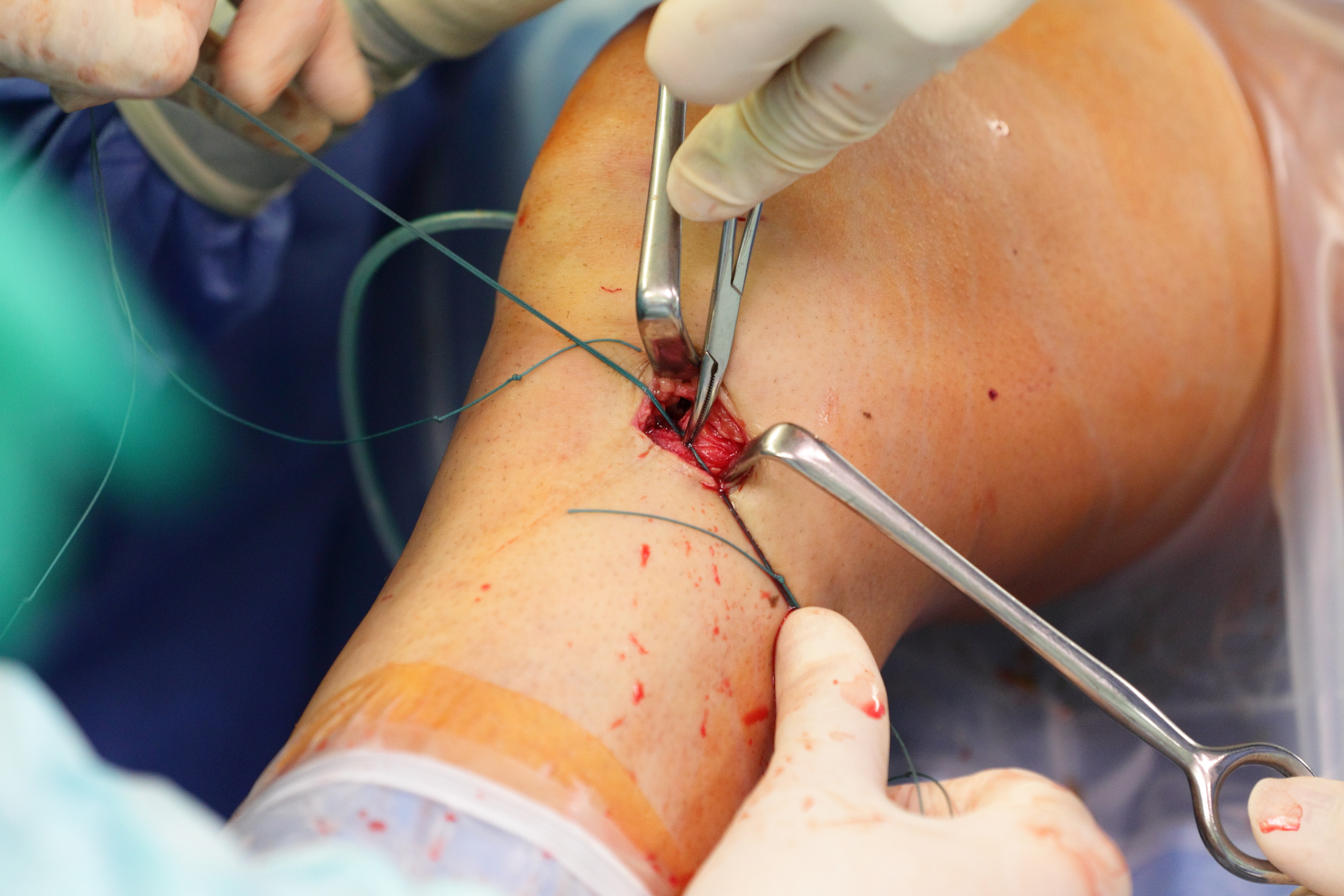

Supplement: Supplementary file 3 — Figure SC. Knotting the Mersilene threads over the tibial bone bridge. (JPG 4293 kb) [file 12891_2018_2271_MOESM3_ESM.jpg]
